# Supplementary material for: XTT assay for detection of bacterial metabolic activity in water-based polyester polyurethane
Source: PLoS One. 2024 Jun 6;19(6):e0303210. doi: 10.1371/journal.pone.0303210 (PMC11156301; doi:10.1371/journal.pone.0303210)
Supplement: S3 File — PDF file with additional figures. (PDF) [file pone.0303210.s003.pdf]

S3 File. Supplementary material

XTT Assay for Detection of Bacterial Metabolic Activity in Water-based Polyester Polyurethane

Nallely Magaña-Montiel[1¶], Luis Felipe Muriel-Millán[1¶] and Liliana Pardo-López[1*]

[1] Departamento de Microbiología Molecular, Instituto de Biotecnología, UNAM, Av. Universidad #2001, Col. Chamilpa, 62210 Cuernavaca, Morelos, México.

* Corresponding author

E-mail: liliana.pardo@ibt.unam.mx (L P-L)

¶ These authors contributed equally to this work.

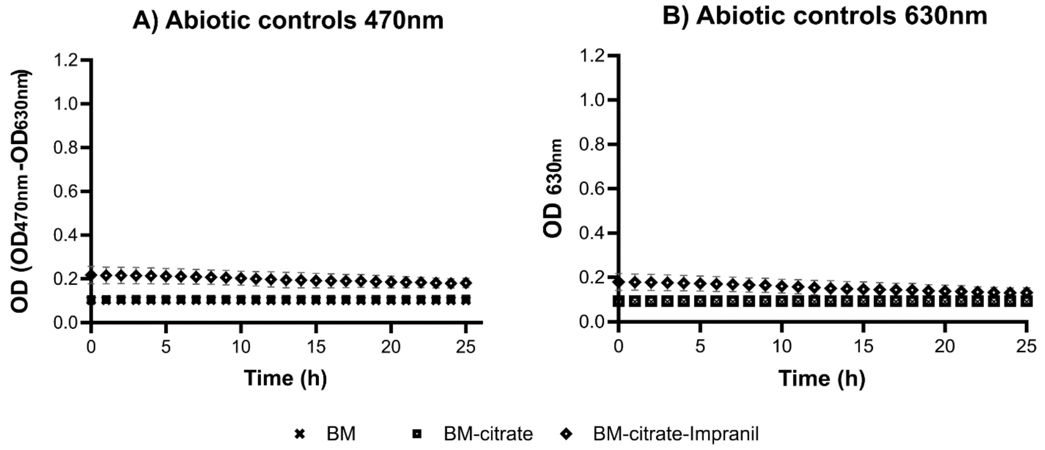

Figure S1. Abiotic controls (OD630nm and OD470nm) for 25 hours (30°C and 180 rpm) in microplate for BM (Basal mineral medium supplemented with Instant Ocean 0.06 g·L^{-1}); Citrate (BM with citrate 20 mM); and Impranil + Citrate (BM supplemented with citrate 20 mM and Impranil 1 mg·mL^{-1}). The data are the mean of the corrected absorbance (OD470-OD630) of three independent experiments performed in duplicate. Error bars; SD.

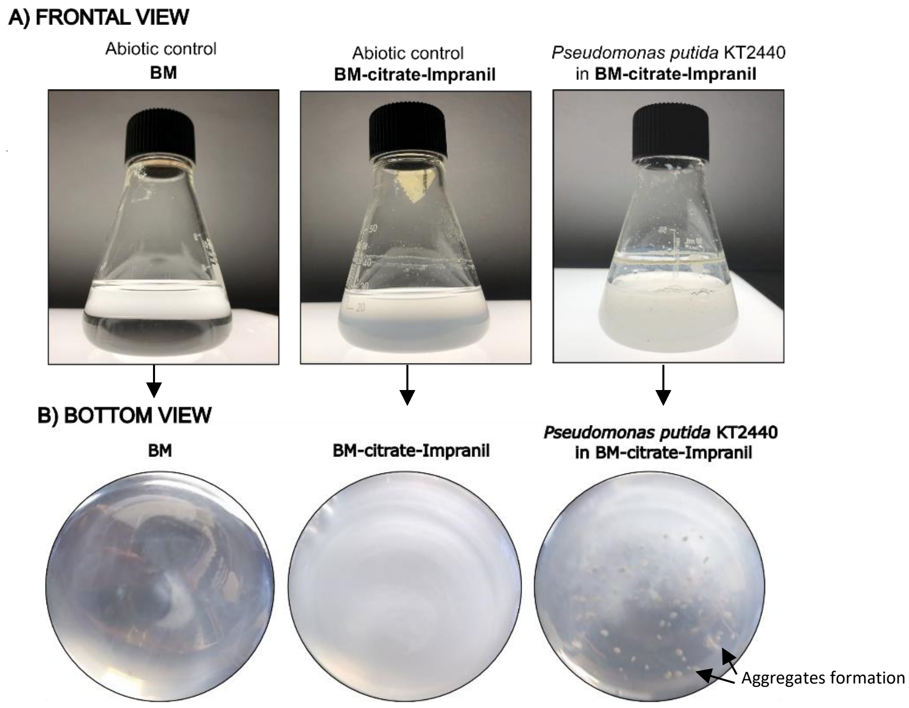

Figure S2. Cells in liquid culture supplemented with Impranil also tend to form aggregates in Erlenmeyer flasks. A) Frontal view of BM media (abiotic control) with no carbon source, BM-citrate-Impranil abiotic control and *P. putida* KT2440 culture in BM-citrate-Impranil. B) Bottom view of Erlenmeyer flasks of BM abiotic control, BM-citrate-Impranil abiotic control; and *P. putida* KT2440 in BM-citrate-Impranil (note the arrows pointing to aggregates dispersed in the culture medium). All treatments are shown after 24 h of incubation at 30°C and 180 rpm in 50-mL Erlenmeyer flasks.

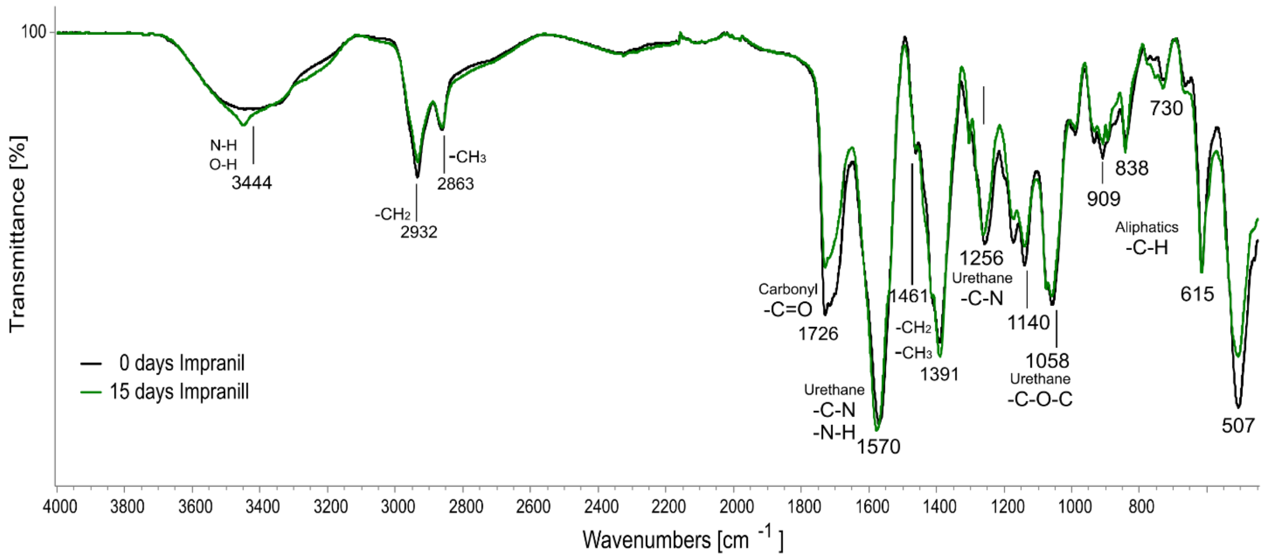

Fig S3. FTIR spectra of Impranil abiotic control for 0 and 15 days of incubation do not show significant changes in functional groups. The FTIR spectra show the average of three biological replicates for each sampling day averaged with Spectragryph software.

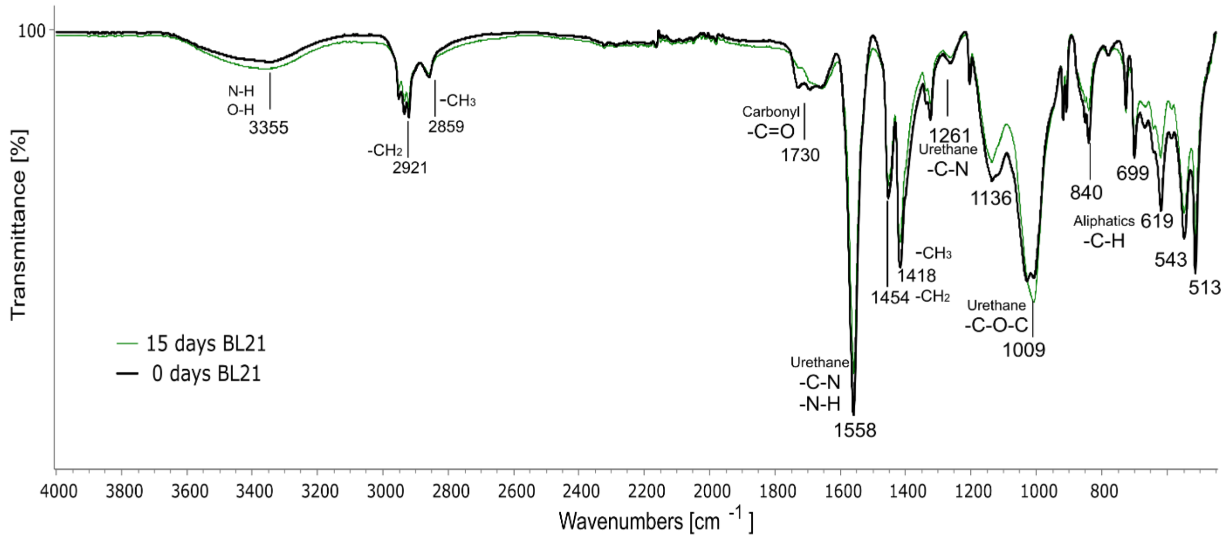

Fig S4. FTIR spectra of *E. coli* BL21 for 0 and 15 days of incubation do not show significant changes in functional groups. The FTIR spectra show the average of three biological replicates for each sampling day averaged with Spectragryph software.

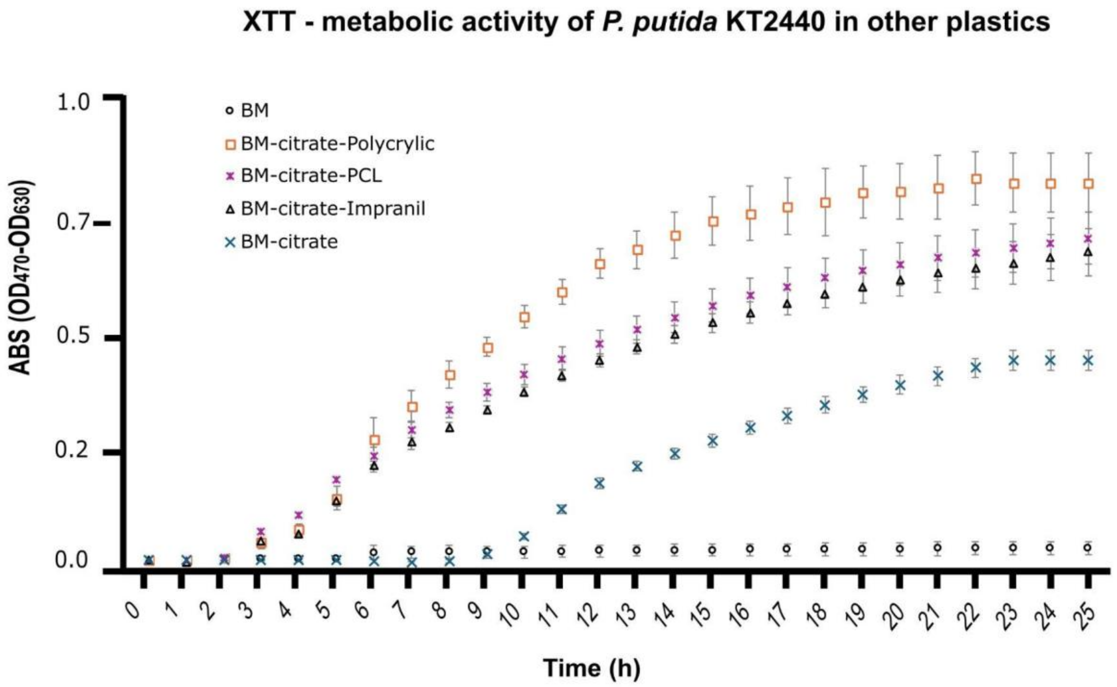

Fig S5. Kinetics of orange-colored formazan production by *P. putida* KT2440 in other types of plastic substrates. *P. putid*a KT2440 shows higher metabolic activity in the presence of plastics than when grown with citrate as a sole carbon source. 50 µL of XTT (2 mg·mL^{-1}) were added to the cultures in microplates of *P. putida* KT2440 in BM: Basal mineral medium with no carbon source, BM-citrate: BM supplemented with 10 mM sodium citrate, BM-citrate-Polycrylic: BM-citrate supplemented with Polycrylic (1 mg·mL^{-1}), BM-citrate-PCL: BM-citrate supplemented with PCL (1 mg·mL^{-1}), and BM-citrate-Impranil: BM-citrate supplemented with Impranil (1 mg·mL^{-1}). The data are the mean of the corrected absorbance (OD470-OD630) of three independent experiments performed in duplicate. Error bars; SD.
