# Supplementary material for: XTT assay for detection of bacterial metabolic activity in water-based polyester polyurethane
Source: PLoS One. 2024 Jun 6;19(6):e0303210. doi: 10.1371/journal.pone.0303210 (PMC11156301; doi:10.1371/journal.pone.0303210)
Supplement: S1 File — PDF file that contains step by step how to perform this experiment and tips for adapting it to other culture conditions. The protocol described in this peer-reviewed article, published on protocols.io, dx.doi.org/10.17504/protocols.io.4r3l27zbjg1y/v1, included for printing. (PDF) [file pone.0303210.s001.pdf]

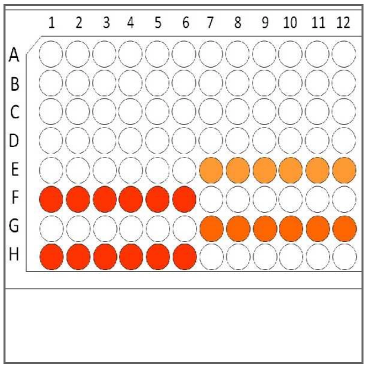


OCT 04, 2023

OPEN ACCESS

**DOI:**
dx.doi.org/10.17504/protocols.io.4r3l27zbjg1y/v1

**Protocol Citation:** Nallely Magaña-Montiel, Luis F Muriel-Millán, Liliana Pardo-López 2023. XTT Assay for Detection of Bacterial Metabolic Activity in water-based Polyester Polyurethane . **protocols.io** https://dx.doi.org/10.17504/protocols.io.4r3l27zbjg1y/v1

**MANUSCRIPT CITATION:**

**License:** This is an open access protocol distributed under the terms of the Creative Commons Attribution License, which permits unrestricted use, distribution, and reproduction in any medium, provided the original author and source are credited

# XTT Assay for Detection of Bacterial Metabolic Activity in water-based Polyester Polyurethane

Nallely Magaña-Montiel[1], Luis F Muriel-Millán[1], Liliana Pardo-López[1]

[1]Departamento de Microbiología Molecular, Instituto de Biotecnología, UNAM, Av. Universidad #2001, Col. Chamilpa, 62210 Cuernavaca, Morelos, Mexico

Nallely Magaña-Montiel: nallely.magana@ibt.unam.mx
Luis F Muriel-Millán: luis.muriel@ibt.unam.mx
Liliana Pardo-López: liliana.pardo@ibt.unam.mx

Study of plastic biodegradation by marine bacteria from the Gulf of Mexico

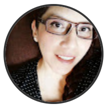

Nallely Magaña-Montiel

## ABSTRACT

In microbial biodegradation assays, the detection of bacterial growth in water-based plastic dispersions can be difficult to measure using traditional methods because of the turbidity of culture media and the formation of flocculi. Here, we present a protocol for the detection of bacterial growth in Impranil®DLN, a polyester polyurethane (PU) water-based dispersion. By measuring bacterial metabolic activity, as an indicator of cell viability with the water-soluble 2, 3-bis [2-methyloxy-4-nitro-5-sulfophenyl]-2H-tetrazolium-5-carboxanilide (XTT) salt. Viable growing cells, i.e., those cells that can utilize PU as a carbon source, will reduce the yellow-colored XTT to a water-soluble orange formazan by the action of dehydrogenase enzymes of the respiratory chain. For the standardization of the protocol, we used *Pseudomonas putida* KT2440 and *Escherichia coli* BL21 strains as positive and negative controls, respectively. We determined the metabolic activity of the strains grown with citrate or both citrate and impranil as carbon sources. P. putida KT2440 showed higher XTT-detected metabolic activity in the presence of PU than when it was grown only with citrate, indicating that the strain also used PU as a carbon source. In contrast, the negative control did not show differences in metabolic activity between the growth conditions. Our protocol can be adapted to different bacterial strains and culture media.

## IMAGE ATTRIBUTION

Laboratory of Marine Biotechnology, Institute of Biotechnology (IBT) UNAM

**Protocol status:** Working
We use this protocol and it's working

**Created:** Mar 22, 2023

**Last Modified:** Oct 04, 2023

**PROTOCOL integer ID:** 79267

**Keywords:** XTT, plastic biodegradation, marine bacteria, Gulf of Mexico, polyurethane

**Funders Acknowledgement:**
Conacyt PhD Scollarship
Grant ID: 417065
PAPIIT-DGAPA
Grant ID: IG200223

## GUIDELINES

### Practical considerations

- The assay can be adapted for other bacterial strains.
- Also, you can use an automated microplate spectrophotometer to directly incubate and measure the transformation of XTT to formazan continuously (i.e., every hour).
- Glassware preparation: before use in this procedure, clean glassware is washed with the solvents to extract contaminants and avoid plastic residues.

MATERIALS

**LEGEND**

[1] for preparation of bacterial culture

[2] for prior preparation of glass material

[3] for XTT assay

**Reusable materials:**

1. Erlenmeyer 50 mL flasks[1]
2. Erlenmeyer 250 mL flasks[1]
3. 50 mL measuring cylinders[1]
4. Glass container for storing solution 1:1 Methanol:Chloroform[2]
5. Bacteriological loops[1]
6. 10 mL pyrex tubes[1]
7. 50 mL graduated cylinder[1]
8. Ice buckets[1]

**Disposable materials:**

1. Sterile 96 well culture microplates[3]
2. Sterile polystyrene petri dishes[1]
3. Sterile 50 mL polypropylene centrifuge tubes[1]
4. Neoprene & Nitrile Chemical resistant Gloves[2]
5. Pipette tips[1,3]
6. Nitrile gloves[1,3]

**Equipment:**

1. Multichannel pipette 20-200 µL with 12 chanels[3]
2. Fume hood[2]
3. Digital clock[1,3]
4. Vortex[1,3]
5. Epoch™ 2 Microplate Spectrophotometer Biotek®[1,3]
6. Centrifuge 5415 R Eppendorf[1]
7. Incubated shaker Lab Companion[1]

**Chemicals:**

1. XTT sodium salt powder (Sigma-Aldrich) Catalog #X4626[3]
2. Basal mineral medium (BM) (Composition in step 8)[1]
3. Impranil®DLN[1,3]
4. Luria-Bertani (LB) agar (NaCl 10 g•$L^{-1}$, peptone 10 g• $L^{-1}$ and yeast extract 5 g •$L^{-1}$, ACS grade)[1]
5. Chloroform ACS grade[2]
6. Methanol ACS grade[2]
7. $MgSO_4$ ACS grade[1]
8. Sodium citrate ACS grade[1,3]

**Strains:**

1. *Pseudomonas putida* KT2440 (positive control)[1,3]
2. *Escherichia coli* BL21 (negative control)[1,3]

**Software:**

1. Microsoft excel[3]
2. Gen5 Data Analysis Software from Epoch™ 2 [3]

PROTOCOL MATERIALS

XTT sodium salt **Merck MilliporeSigma (Sigma-Aldrich) Catalog #X4626** Step 9

SAFETY WARNINGS

**This procedure involves the use of hazardous chemicals (chloroform and methanol) for the glassware preparation.**
**XTT contains no substances with occupational exposure limit values.**

1. Read the corresponding safety data sheets for each chemical in the procedure.
2. Use personal protective equipment throughout the procedure: nitrile gloves, wear a lab coat and safety glasses.
3. Dispose of all chemical waste in appropriately labeled containers.

BEFORE START INSTRUCTIONS

- **Washing glassware**
  All glassware should be washed twice using 2 mL of methanol-chloroform mixture (1:1) and allowed to dry in a fume hood.

## Preparation of bacterial strains

1 Scrape some of the frozen surface of the glycerol stock using a sterile loop and streak the bacteria onto a Luria-Bertani (LB) agar plate.

2 Incubate the culture at 30 °C during 24:00:00 1d

Note

Use adecuate incubation temperature for your strain.

3 Take one isolated colony and inoculate it into 5 mL LB broth.

4 Incubate Overnight at 30 °C with shaking at 180 rpm .

4.1 Measure the optical density (OD) at 600 nm of cultures using a spectrophotometer.

Note

If needed, prepare a dilution of the culture (e.g., $10^{-1}$) using fresh LB broth and measure its $OD_{600}$. The $OD_{600}$ of the original culture is calculated by multiplying the obtained OD by the dilution factor.

5 Inoculate 250-mL flasks containing 50 mL of Basal Medium standardize supplemented with Instant Ocean Sea Salt (0.06 $g \cdot L^{-1}$) (BM), peptone (10 $g \cdot L^{-1}$) and yeast extract (5 $g \cdot L^{-1}$) (BM-PY broth) with an aliquot of the previous culture to obtain an $OD_{600}$ of approximately 0.1.

Note

Our research group uses marine salts to standardize the protocol and screen marine bacteria for subsequent toxicity assays of bacterial culture in zebrafish embryos. For your specific requirements, feel free to use an appropriate minimal medium and carbon source. Composition of Basal Medium in $g \cdot L^{-1}$: 0.8 $K2HPO_4$, 0.2 $KH_2PO_4$, 0.3 $NH_4Cl$, 0.19 $Na_2SO_4$, 0.07 $CaCl_2$, 0.005 $FeSO_4 \cdot 7H_2O$, 0.16 $MgCl_2$, and 0.0002 $Na_2MoO_4$ (all J.T. Baker® ACS grade).

CITATION

Muriel-Millán LF, Rodríguez-Mejía JL, Godoy-Lozano EE, Rivera-Gómez N, Gutierrez-Rios RM, Morales-Guzmán D, Trejo-Hernández MR, Estradas-Romero A, Pardo-López L. (2019). Functional and Genomic Characterization of a Pseudomonas aeruginosa Strain Isolated From the Southwestern Gulf of Mexico Reveals an Enhanced Adaptation for Long-Chain Alkane Degradation.. Front. Mar. Sci. 6:572.

LINK

https://doi.org/10.3389/fmars.2019.00572

6 Incubate the new culture at 180 rpm, 30°C for the time needed to reach the exponential growth phase: 4h approximately 04:00:00 (see the note below).

Note

This 4 hour timeframe applies to our experimental and cultural conditions. It is important to be knowledgeable of the exponential growth rate of your bacterial strains.

7 Collect the cells by centrifugation at 6000 rpm, 4°C, 00:20:00 and discard the supernatant. 20m

7.1 Wash twice the cellular pellet by suspension in 20 mL of sterile 10mM $MgSO_4$ followed by centrifugation at 6000 rpm, 4°C, 00:10:00 to remove all traces of the old culture medium. 10m

7.2 Resuspend the cells in 5 mL of fresh sterile 10mM $MgSO_4$ and reserve to be used as inoculum for the next steps. Keep the washed cells on ice to facilitate their handling and preparation.

## Culture media preparation

8 The strains' ability to grow using the commercial PU coating Impranil® DLN as a carbon source is evaluated in Basal Medium, which is always supplemented with Instant Ocean Sea Salt (0.06 g•$L^{-1}$).

9 **XTT solution**

Prepare a solution of XTT sodium salt **Merck MilliporeSigma (Sigma-Aldrich) Catalog #X4626** at 2 mg•$mL^{-1}$ in BM.

10 **BM-citrate solution**

Prepare a solution of 20 mM sodium citrate in BM.

Note

The presence of easily metabolizable carbon sources such as citrate has been shown to promote the degradation of xenobiotic compounds (Fonseca 2011, Johnsen et al. 2002).

CITATION

Johnsen Anders R., Bendixen Karen, Karlson Ulrich (2002). Detection of Microbial Growth on Polycyclic Aromatic Hydrocarbons in Microtiter Plates by Using the Respiration Indicator WST-1. Applied and Environmental Microbiology.

LINK

doi: 10.1128/AEM.68.6.2683-2689.2002

11 **BM-citrate-Impranil solution**

Prepare a BM-citrate solution added with Impranil®DLN (1 mg•mL$^{-1}$)

## XTT experiment for detection of bacterial growth

12 **XTT experiment** standardization

Add 150 µL of the BM solution and 50 µL of the XTT solution into a 96-well microplate and measure the UV-Vis spectrum in a range of 300 to 700 nm with a microplate spectrophotometer. Also, obtain the spectrum of BM-citrate and BM-citrate-Impranil in the same UV-Vis range. Perform in triplicate.

Note

Perform experiment standardization for each condition, i.e., for each medium. The signal intensity is subject to several parameters, including concentration of formazan salts, incubation time, number of viable cells, and cellular metabolic activity. Optimization of test conditions requires consideration of all these parameters (Ghasemi et al. 2021, Riss et al. 2023).

CITATION

Ghasemi M, Turnbull T, Sebastian S, Kempson I (2021). The MTT Assay: Utility, Limitations, Pitfalls, and Interpretation in Bulk and Single-Cell Analysis..

LINK

https://doi.org/10.3390/ijms222312827

CITATION

Terry L Riss, PhD, Richard A Moravec, BS, Andrew L Niles, MS, Sarah Duellman, Hélène A Benink, Tracy J Worzella, and Lisa Minor (2013). Cell Viability Assays. Assay Guidance Manual .

LINK

https://www.ncbi.nlm.nih.gov/books/NBK144065/

13

**To obtain the maximum absorbance range of XTT with viable cells:**

Prepare *Pseudomonas putida* KT2440 (positive control) cultures in BM-citrate, BM-citrate-Impranil, and BM without any carbon source (biotic control), by inoculating 20 mL of each medium on 50-mL flasks up to obtain an $OD_{600}$ approximately of 0.3 (~1x $10^8$ cells) in triplicate.

To obtain the maximum absorbance range, add 150 µL of each culture medium of *Pseudomonas putida* KT2440 and 50 µL of the XTT solution into a 96-well microplate and measure UV-Vis spectrum in a range of 300 to 700 nm with a microplate spectrophotometer, immediately after the addition of XTT and again after 1-3 hours of incubation at 180 rpm, 30°C in dark.

**Note**

The assay can be adapted for other bacterial strains.
Also, you can use an automated microplate spectrophotometer to directly incubate and measure the transformation of XTT to formazan continuously (i.e. every hour).

Expected result

Cells with metabolic activity reduce XTT (yellow) to formazan (orange), which has a wavelength range of maximum absorbance between 450-500 nm (Cell Counting & Health Analysis, 2023; Maldonado et al. 2017).

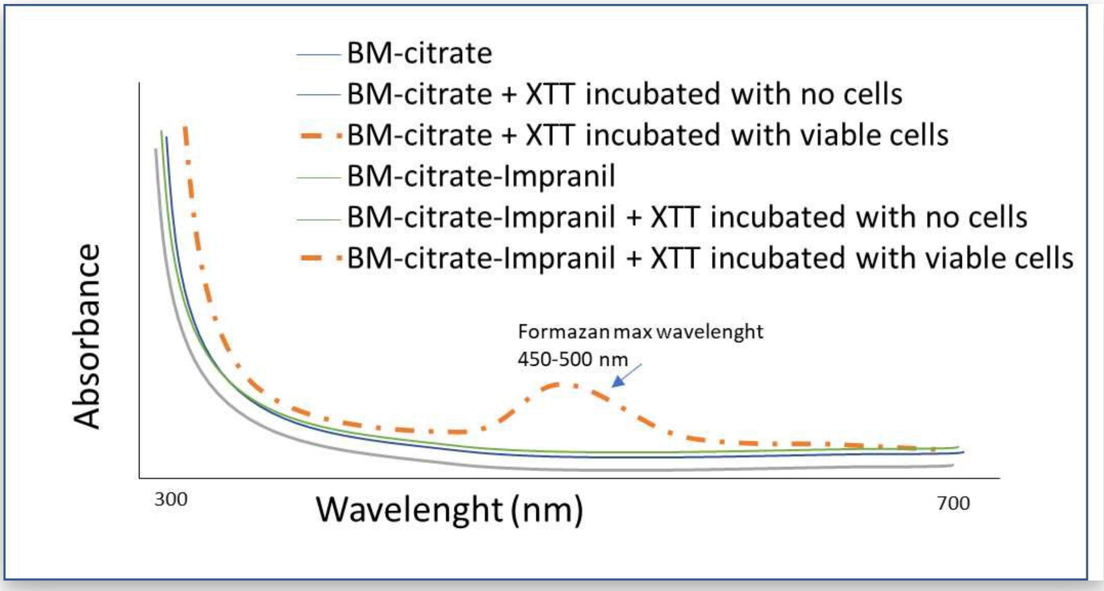


CITATION

Maldonado Cubas, Juan & Casañas Pimentel, Rocio & Merlin, Iván & San Martín-Martínez, Eduardo. (2018). La espectroscopia UV-Vis en la evaluación de la viabilidad de células de cáncer de mama. Lat. Am. J. Phys. Educ..

LINK

https://www.researchgate.net/publication/340915352_La_espectroscopia_UV-Vis_en_la_evaluacion_de_la_viabilidad_de_celulas_de_canc

CITATION

Cell Counting & Health Analysis, Merck guides, online 2023 (2023). Cell Viability and Proliferation XTT Assay Protocol Guide.

LINK

https://www.sigmaaldrich.com/MX/es/technical-documents/protocol/cell-culture-and-cell-culture-analysis/cell-counting-and-health-

## Detection of Bacterial Growth in Polyester Polyurethane

14 To evaluate microbial growth, measure the absorbance of each treatment every hour with an automated microplate spectrophotometer at $Abs_{max}$ (range of 450nm - 500nm) with a reference wavelength of 630nm immediately after the addition of XTT and again every hour.

**Note**

The use of a reference wavelength considerably reduces the noise of the particles in the medium (background subtraction at 630–690 nm). Also, subtract the absorbance at time zero ($Abs_i$) from subsequent readings to obtain the change in absorbance.

$$Absorbance= [Abs_{max} - Abs_{630}] - Abs_i$$

Culture media:

| A | B | C | D | E |
|---|---|---|---|---|
| | Abiotic control | Pos. control | Neg. control | Test microorganism |
| BM | x | x | x | x |
| BM-citrate | x | x | x | x |
| BM-citrate-Impranil | x | x | x | x |

"x" indicates that must be included.

**Note**

Note that BM in positive control, negative control, and test microorganism correspond to biotic controls without any carbon source.

15 Add 150 µL of the different cultures into a 96-well microplate containing 50 µL of XTT in each well.

We tested the metabolic activity of *P. putida* KT2440 (Franklin et al. 1981) and *E. coli* BL21 as positive and negative controls, respectively.

CITATION

F. C. Franklin, M. Bagdasarian, M. M. Bagdasarian, and K. N. Timmis (1981). Molecular and functional analysis of the TOL plasmid pWWO from Pseudomonas putida and cloning of genes for the entire regulated aromatic ring meta cleavage pathway.. Proc.NatLAcad.Sci.USA.

LINK

https://doi.org/10.1073/pnas.78.12.7458

Note

The assay can be adapted for other bacterial strains.

16 Immediately after XTT addition, incubate the plate 24:00:00 at 30. °C 1d

16.1

To evaluate microbial growth, measure absorbance every hour with an automated microplate spectrophotometer reader EPOCH2 (BioTek Instruments Inc.) both at 470nm and 630nm (reference wavelength for background subtraction).

**Expected result**

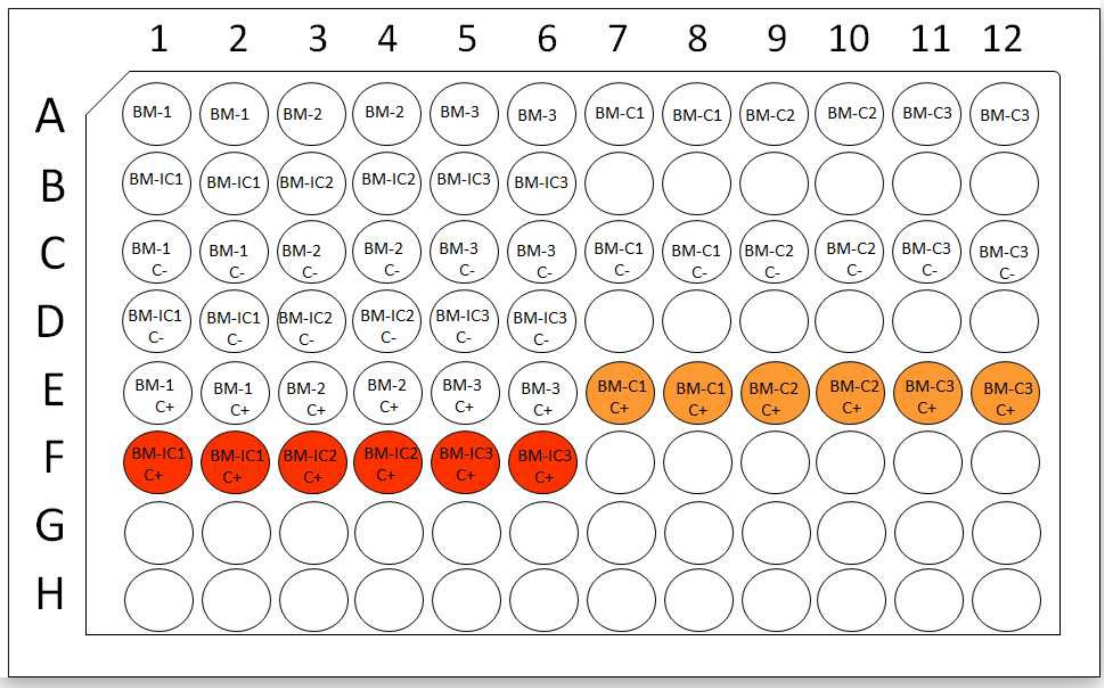


Expected results for XTT assay. Viable cells transform the yellow XTT to formazan (orange). The signal intensity depends both on the number of viable cells and the cellular metabolic activity. BM-C: BM-citrate, BM-IC: BM-citrate-Impranil, C-: negative control, C+: positive control. The numbers indicate biological replicates each one performed in duplicate (technical replicates).
